# Case for diagnosis. Multiple nodules on the scrotum^☆^

**DOI:** 10.1016/j.abd.2022.10.012

**Published:** 2023-08-09

**Authors:** Natsuko Matsumura, Toshiyuki Yamamoto

**Affiliations:** Department of Dermatology, Fukushima Medical University, Fukushima, Japan

Dear Editor,

An 83-year-old male visited us complaining of multiple nodules on the scrotum that first appeared 20 years previously. They had been increasing in size and recently started to bleed easily. Physical examination revealed 2 exophytic and pedunculated red nodules sized 25 × 25 mm and 13 × 13 mm, which protruded from both sides of the scrotum (Fig. 1). He has a history of prostate cancer, obstructive hypertrophic cardiomyopathy, aortic regurgitation, chronic atrial fibrillation, chronic renal failure, and submucosal tumors of the esophagus. A biopsy specimen showed upwardly protruding tumors with acanthosis and papillomatosis (Fig. 2). The dermal papillae were covered by numerous foamy histiocytes and hyperplasia of capillaries (Fig. 3). The foamy cells were positive for Periodic Acid Schiff and CD68 antigen (Fig. 4).

**Figure 1:**
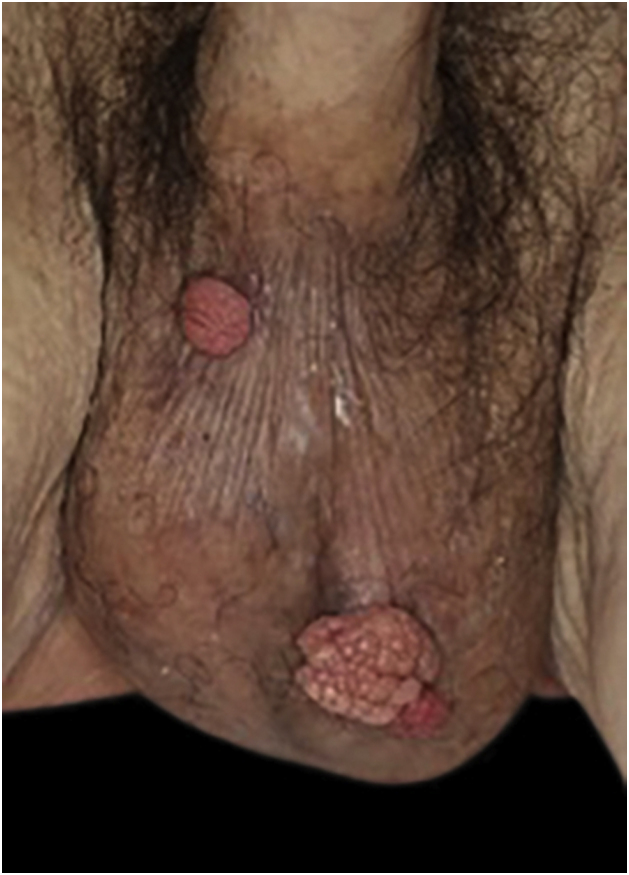
Well-circumscribed, exophytic and pedunculated nodules on the scrotum

**Figure 2:**
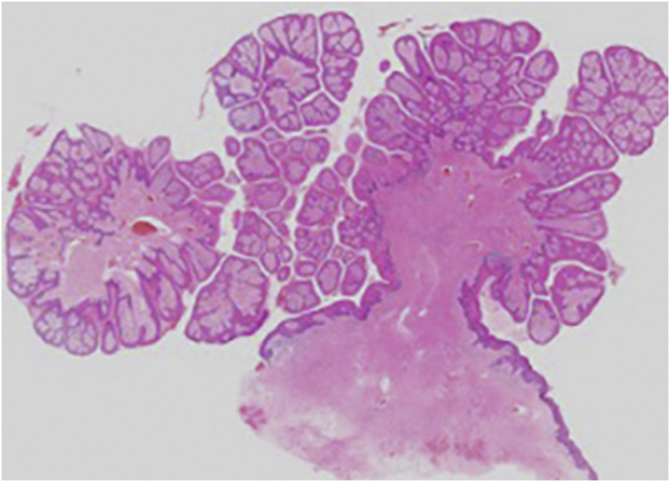
Histopathological findings showed upwardly protruding tumors with acanthosis and papillomatosis

**Figure 3:**
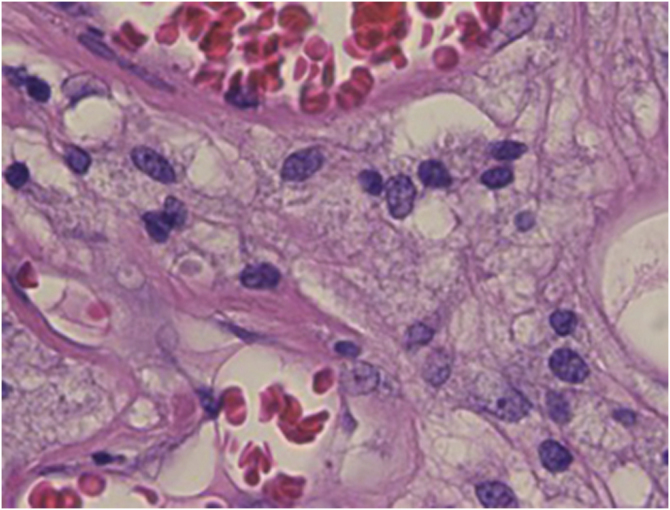
Detail of histopathology: The dermal papillae are covered by numerous foamy histiocytes with increased number of capillaries

**Figure 4:**
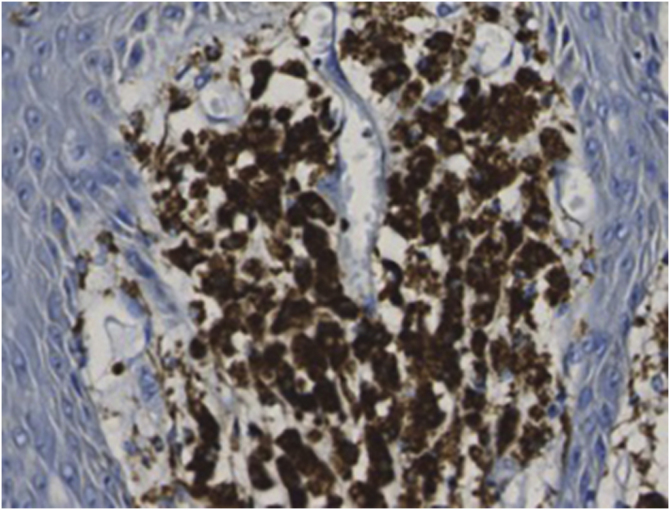
Foamy macrophages in the papillary dermis showing CD68 positivity

## What is your diagnosis?

a) Viral wart;
b) Condyloma acuminatum;
c) Verruciform xanthoma;
d) Adult xanthogranuloma.

## Discussion

From the histopathological findings, the nodules were diagnosed as Verruciform Xanthoma (VX). Xanthogranuloma was excluded because Touton-type giant cells were not observed. Both of the nodules were surgically removed, and they showed the same histopathological features. No recurrence had been observed during 1-year follow-up.

VX occurs mostly in the oral cavity^1^; however, the genital area has also been involved (verruciform genital-associated xanthoma).^2^ Clinically, VXs have a similar appearance to condyloma acuminatum or verruca vulgaris. The histopathological examination demonstrated verrucous hyperplasia of the epidermis and a variable number of foamy cells within the dermal papillae. As far as we reviewed, only 6 cases of multiple VXs on the genital area have been reported including the present case (Table 1).^3^, ^4^, ^5^, ^6^, ^7^ Those 6 cases consisted of 4 males and 2 females, and the mean age was 53 years old (range 29‒83 years). The size of the lesions ranged from 3 to 25 mm. The number of nodule was 2 in our case, whereas numerous nodules were observed in 1 case.^3^ Pruritus was observed in 2 cases, whereas others were asymptomatic. In 1 case, VX occurred at the site of the skin graft due to necrotizing fasciitis.^5^

**Table 1:** Reports focusing on verruciform xanthoma in multiple sites on the genital area

Patients	Age/Gen	Duration of illness	Clinical hypothesis	Size (mm)	Location	Symptoms	Progress	Trigger	HPV
1^3^	29F	17-yr	Condyloma	ND	Vulva	None	Not change	ND	ND
2^4^	42F	20-yr	ND	3 to 25	Vulva	ND	Increasing	ND	Negative
3^5^	38M	2.5-mo	Necrotizing fasciitis	8 × 5	Penis and perineum	None	Enlarging	Skin grafting	ND
4^6^	63M	4-yr	Condyloma	10 to 15	Scrotum	Pruritic	Increasing	ND	ND
5^7^	67M	4-yr	ND	25	Scrotum	ND	Increasing	ND	HPV 6a
Our case	83M	20-yr	Condyloma	13 to 25	Scrotum	Pruritic	Increasing/bleeding	External factor	Negative

ND, Not Described.

Although the cause of VX is unknown, Zegarelli et al. suggested that VX results from degenerative changes in the epidermis with a subsequent nonspecific histiocytic response.^8^ The damage to the epithelium could trigger the following cascade: 1) Entrapment of epithelial cells in the papillary dermis, 2) Subsequent degeneration of these cells and lipid formation, 3) Engulfment of released lipids by macrophages, and 4) Accumulation of foam cells between the rete ridges. On the other hand, it was speculated that the Human Papillomavirus (HPV) was involved in the pathogenesis of VX. Khaskhely et al. reported VX in which HPV type 6a DNA was detected in the lesional tissues by Polymerase Chain Reaction (PCR) and sequence analysis.^7^ By contrast, another study examined HPV types 6, 11, 16, 18, 31, 33, and 35 by PCR, which were all negative.^9^ In the present case, PCR amplification of HPV including serotypes 6, 11, 16, 18, 31, 33, 35, 52b, and 58 was performed, with all negative results. Therefore, further studies of the etiology of VX are necessary.

## Financial support

None declared.

## Authors' contributions

Natsuko Matsumura: Collection, analysis, and interpretation of data; drafting and editing of the manuscript; critical review of the literature.

Toshiyuki Yamamoto: Design and planning of the study; editing and final approvement of the manuscript.

## Conflicts of interest

None declared.
